# The Prophylactic Value of Protective Injections

**Published:** 1919-01-18

**Authors:** 


### INFLUENZA AND IMMUNITY.

### The Prophylactic Value of Protective Injections.

One has read, of late, reports of so many observations, both laboratory and clinical, and so much theoretical and speculative discussion concerning this formerly very commonplace disease, that apology must be made for heaping even one further remnant into the confusion; and yet, having noted the main conclusions drawn at the meeting of the Royal Society of Medicine meeting on November 13, one is inclined to doubt whether the situation presents so much uncertainty after all.

### Fostered by War Conditions.

Put briefly, it would seem that we have to deal with repeated infections by the usual catarrhal bacteria, whose virulence has been carefully fostered and increased by the unavoidable conditions of the war. Warmth, moisture, and repeated transference to fresh soil combine, within limits, to produce their ideal reproductive and toxic strength. Modern requirements have called for the massing of human beings in a thousand and one ways, modern circumstance has been entirely opposed to really efficient prophylactic measures and the result inevitable. Also, in the laboratory, the disease, according to their findings, seems hardly to fulfil its early promise of novelty, and one must consider its unbelievably rapid spread, its tremendous range, embracing at one time Europe, India, and the Americas, and its curiously fluctuating intensity in order fully to appreciate its claims to attention.

So may we proceed plausibly enough, but the next steps lead to discrepancies. To mention one of the many minor difficulties : the actual dates at which waves of maximum intensity have appeared and the recognised seasonal incidence have in no way corresponded. Whether the divergence be due to overlapping or summation of waves starting from different quarters or to the increased vigour of the organisms, giving them new-found climatic and seasonal freedom is very hard to decide. Possibly both factors come into play, but, in this connection, and having a very distinct influence upon the run of these waves, must be considered the question of acquired immunity.

Opinions on the subject appear to differ widely. Of the pre-war disease we were told that, clinically, one or even two attacks did not protect; but at that time one can say that people were rarely exposed to a second or fresh epidemic until perhaps a year later; then they again developed symptoms, their immune period being too short to be effective. The conditions changed. Men were brought up against an absolutely new and fresh strain of organisms within a few weeks of their first infection, possibly during convalescence even. Then an immunity, however short, must have had a very marked bearing on the case incidence, and that this influence is very real and very active is well brought out in the case of a ship's company of some seven hundred men. There seems no room for doubting that many of these men acquired considerable, if not absolute, powers of resistance to repeated contacts with the germs, and although, as indicated later, one has no proof of specific antitoxic powers towards influenza or its modern prototype, it is quite certain that the men mixed freely with the inhabitants of infected areas and yet remained in full health.

### Outbreaks on Board Ship.

A series of voyages covered the whole of the epidemic period of the past year and, being as long as ten to sixteen days out of touch with land, the result of each spell in harbour with its possible infections could be very readily noticed. Sufficient time elapsed for a full incubation period and one may reasonably consider the ship as completely isolated when in contact with the sea and the ocean winds alone. Of the local conditions, the most noteworthy are, firstly, the continual between-decks war with arid humidity while at sea; secondly, the relative crowding of the man in an all too insufficient air-space (according to shore standards at least); and, thirdly, the high proportion of "hostilities only" ratings, representing an average and varied assembly when compared with the fit, standard, lifelong sailor.

Three separate outbreaks occurred and may be outlined here.

The first contact was made in the North of Scotland early in the year. A high percentage of the company showed catarrhal symptoms, but none were severe enough to find their way to the hospital ship. In every respect it appeared to be an active "pre-war" influenza. A necessarily limited bacteriological examination was made in some twenty-five cases, and cultures on blood agar gave strikingly similar results with all of them. In each was found a hæmolytic streptococcus, and, in more than half, a small gram-negative bacillus undistinguishable, without series culture tests, from *B. Influenza.* Of the men attacked, one could say that they were mainly the more obviously weak or catarrhal subjects, such as adenoid mouth breathers, mild bronchitis, etc. No serious complications ensued, and within a few weeks of work in the North Sea all traces of the outbreak had vanished.

We now come to the second contact. No further probability of infection occurred until May, when the ship was docked at a Midlands port where the shore epidemic was at its height. Within a week, and with an incubation period of two to three days, another sixty cases had appeared. The virulence had undoubtedly increased and, although the character of the initial symptoms had not changed, the definite subdivision into meningeal, gastric, and bronchitic types of extension was very marked. Bacteriologically an exact repetition of results was obtained. Case details are redundant here, but it is noteworthy that only two men who had been previously attacked were to be found in this batch, and in neither case had their Scotch infection been severe enough to put them on to the sick list.

The third invasion occurred two months later, when a few days were spent in an American port, then in the throes of its first severe outbreak. Within three days cases began to appear. The onset was typical as before, but followed this time by the acutest complications. Early departure necessitated the retention on board of all but one of the invalids, and, with septicæmia and pneumonia not uncommon, the greatest difficulty was experienced in treating and nursing the men at sea. Two terminated fatally within a week, and several more ultimately died in British hospitals at the end of the voyage. This time the streptococcus was particularly obvious in culture, and in one case of septicæmia it was actually grown from the patients' blood, while similar reports were obtained later upon cases sent to hospitals ashore. And here again one must remark that only three were re-infectious, and these three men developed no complications whatever, but showed a normal tempera-

**Influenza and Immunity**—(*continued*).

ture within five days. Also, with regard to this return voyage, a particularly interesting comparison may be drawn. A troop transport sailing at the same time and in company, with men infected in the same area but for the first time, showed, in spite of far better hygienic surroundings, a casualty list proportionately seven times heavier than our own.

Even up to this point the developed immunity seems quite definite, but the sequel is important and gives further proof in that, four months later, although several times in contact with the disease in Great Britain, America, and Canada, only six catarrhal cases occurred in the ship, and these only very mild in degree. For all practical purposes the ship's company can be taken as the same throughout, very few changes in personnel taking place and nearly all the hospital cases being returned to the ship after convalescence.

The Chief Deduction.

It is beside the point here to describe prophylactic measures, attempts at isolation, disinfection, and the many ways in which the bacteria, once inside the ship, were counter-attacked, for it is to the main issue that the chief deduction from these notes must lead. Superficial as the bacteriological work in a ship had perforce to be, there was a decided and definite recurrence of the typical pathological streptococci. The antitoxic powers of streptococcic sera are well established. The artificially immunised animal is a common experimental, even commercial entity. Take this organism as the common cause of the severer complications, largely responsible for the high mortality and entirely free from limitations of action generally ascribed to the accompanying bacillus, and it is seen that many of the abnormalities in these outbreaks can be explained as due to our "influenza" being a subacute streptococcal infection. To such we may grant the certainty of some acquired immunity, be the invasion acute or so slight as to pass unnoticed. Working under cover and behind the screen of the coccal predominance we can picture the bacilli, but exactly how *B. Influenza* has changed, if at all, is beyond the scope of such rough notes as these, where the attempt is to point out that immunity is developed to the mixed bacteria as a whole. And to this end one must impress three facts, namely, that many of the ship's cases were definite mixed infections, that the men were again exposed to similar combinations of germs, and that a considerable degree of immunity was at once exhibited.

Doubtless many such experiences might be cited, and, by the time we have the statements of the innumerable officialdoms of the world at war and the true picture of the disease in detail and perspective, it will be more than interesting to learn whether we are to describe the true causes of its abnormalities as complications of circumstance and distribution, disguising the habits of very well-known organisms, or as appearances of new bacterial forms, deserving of special description. Evidence favours the former, though, as yet, incompletely. The final decision must come later, but does not primarily concern us here. There may be bacilli, pneumococci, and streptococci in their many forms, all liable to take action in the human body. More than one authority has denied to these the right of distinctive names and classified them merely as metamorphoses of a common ancestor. But one would, apart from such considerations, call attention to presence amongst us of thousands of people who have gained a marked immunity to influenza attacks. Why should we not make a general attempt to immunise the masses?

It has been done with remarkable success in the case of the typhoid group of infections. These diseases, in their most destructive form, are practically wiped out, and it must be recalled that already many extremely successful protective inoculations have been made against influenza. The details of the preparations employed are not at present well collected and described, but would appear to comprise mixed vaccines derived from the usually recognised catarrhal organisms. It is known that their use in certain well-known schools and Service establishments has led to almost complete protection against the disease.

Such facts can be gleaned from the literature, and, adding the accumulation of such small experiences as here described, there are the strongest indications for a belief in acquired resistance. The combination of proved results from both inoculation and auto-inoculation should make a powerful case for those who would urge and develop the prophylactic value of protective injections. With typhoid they led to one of the great medical victories of our time. May we hope for equal success in the combat with this latest scourge? It would seem to be an attainable ambition and worthy of the highest endeavour.

